# RETRACTION: A Comprehensive Study on the Risk Factors and Pathogen Analysis of Postoperative Wound Infections Following Caesarean Section Procedures

**DOI:** 10.1111/iwj.70607

**Published:** 2025-04-21

## Abstract

**RETRACTION:** DongH. , SongJ. , JiaY. , CuiH. , and ChenX. , “A Comprehensive Study on the Risk Factors and Pathogen Analysis of Postoperative Wound Infections Following Caesarean Section Procedures,” International Wound Journal 21, no. 1 (2024): e14609, 10.1111/iwj.14609.38272798 PMC10801270

The above article, published online on 21 January 2024, in Wiley Online Library (http://onlinelibrary.wiley.com/), has been retracted by agreement between the journal Editor in Chief, Professor Keith Harding; and John Wiley & Sons, Ltd. Following an investigation by the publisher, all parties have concluded that this article was accepted solely on the basis of a compromised peer review process. The editors have therefore decided to retract the article. The authors did not respond to our notice regarding the retraction.

**RETRACTION:** H. Dong , J. Song , Y. Jia , H. Cui , and X. Chen , “A Comprehensive Study on the Risk Factors and Pathogen Analysis of Postoperative Wound Infections Following Caesarean Section Procedures,” International Wound Journal 21, no. 1 (2024): e14609, 10.1111/iwj.14609.38272798 PMC10801270

The above article, published online on 21 January 2024, in Wiley Online Library (http://onlinelibrary.wiley.com/), has been retracted by agreement between the journal Editor in Chief, Professor Keith Harding; and John Wiley & Sons, Ltd. Following an investigation by the publisher, all parties have concluded that this article was accepted solely on the basis of a compromised peer review process. The editors have therefore decided to retract the article. The authors did not respond to our notice regarding the retraction.

